# Predictors of pulmonary tuberculosis treatment outcomes in South Korea: a prospective cohort study, 2005-2012

**DOI:** 10.1186/1471-2334-14-360

**Published:** 2014-07-02

**Authors:** Hongjo Choi, Myungsun Lee, Ray Y Chen, Youngran Kim, Soyoung Yoon, Joon Sung Joh, Seung Kyu Park, Lori E Dodd, Jongseok Lee, Taeksun Song, Ying Cai, Lisa C Goldfeder, Laura E Via, Matthew W Carroll, Clifton E Barry 3rd, Sang-Nae Cho

**Affiliations:** 1 International Tuberculosis Research Center, Changwon, Republic of Korea; 2 BK21PLUS Program in Public Health Sciences, Department of Health Sciences, Graduate School, Korea University, Seoul, Republic of Korea; 3 Tuberculosis Research Section, Laboratory of Clinical Infectious Disease, National Institute of Allergy and Infectious Diseases (NIAID), National Institutes of Health (NIH), Bethesda, Maryland, USA; 4 Department of Internal Medicine, National Medical Center, Seoul, Republic of Korea; 5 National Masan Tuberculosis Hospital, Changwon, Republic of Korea; 6 Biostatistics Research Branch, NIAID, NIH, Bethesda, Maryland, USA; 7 Department of Microbiology and Institute for Immunology and Immunological Diseases, Brain Korea 21 Project for Medical Science, Yonsei University College of Medicine, Seoul, Republic of Korea

**Keywords:** Multidrug-resistant tuberculosis, Tuberculosis, Diabetes, Unfavorable outcome, Long-term follow up, South Korea

## Abstract

**Background:**

Tuberculosis remains an important health concern in many countries. The aim of this study was to identify predictors of unfavorable outcomes at the end of treatment (EOT) and at the end of study (EOS; 40 months after EOT) in South Korea.

**Methods:**

New or previously treated tuberculosis patients were recruited into a prospective observational cohort study at two hospitals in South Korea. To identify predictors of unfavorable outcomes at EOT and EOS, logistic regression analysis was performed.

**Results:**

The proportion of multidrug-resistant tuberculosis (MDR-TB) was 8.2% in new cases and 57.9% in previously treated cases. Of new cases, 68.6% were cured, as were 40.7% of previously treated cases. At EOT, diabetes, ≥3 previous TB episodes, ≥1 significant regimen change, and MDR-TB were significantly associated with treatment failure or death. At EOS, age ≥35, body-mass index (BMI) <18.5, diabetes, and MDR-TB were significantly associated with treatment failure, death, or relapse. Among cases that were cured at EOT, age ≥50 and a BMI <18.5 were associated with subsequent death or relapse during follow-up to EOS. Treatment interruption was associated with service sector employees or laborers, bilateral lesions on chest X-ray, and previous treatment failure or treatment interruption history.

**Conclusions:**

Risk factors for poor treatment outcomes at EOT and EOS include both patient factors (diabetes status, age, BMI) and disease factors (history of multiple previous treatment episodes, MDR-TB). In this longitudinal, observational cohort study, diabetes mellitus and MDR-TB were risk factors for poor treatment outcomes and relapse. Measures to help ensure that the first tuberculosis treatment episode is also the last one may improve treatment outcomes.

**Trial registration:**

ClinicalTrials.gov ID: NCT00341601

## Background

Tuberculosis (TB), a global concern for both developing and developed countries, has recently become more complex due to increasing levels of drug resistance and HIV co-infection [1]. Asian and African countries share the highest burden of tuberculosis, accounting for about 85% of the 8.6 million newly diagnosed TB cases reported globally in 2012 [2]. Efforts to reduce disease burden have been largely focused on improving treatment and diagnosis of patients with active disease [3]. Although HIV co-infection and multidrug-resistant (MDR) TB are major contributors to the global TB epidemic [4], a deeper understanding of other risk factors for poor outcome can suggest interventions that might help reduce morbidity and mortality. Poor socioeconomic status, including poverty, lack of education, and urbanization are known risk factors for active tuberculosis [5,6]. Smoking and other behaviors such as alcohol consumption and drug use are also associated with poor treatment outcomes [7-10]. Clinical characteristics including diabetes, baseline disease severity (on chest X-ray), previous treatment history, and drug-resistance have all been shown to be independent risk factors for poor treatment outcomes in previous studies [11-13].

Few studies have prospectively identified factors associated with long-term prognosis. A retrospective study by Kim et al [14] evaluated long-term prognostic factors among MDR-TB patients and found that having extensively drug resistant (XDR) TB was the strongest predictor of poor outcomes. Two other studies evaluated the characteristics of patients with tuberculosis relapse after treatment completion [15,16]. In one, a prior history of TB treatment was the largest risk factor associated with TB recurrence (HR: 5.2, CI: 1.7-16.2) [16] and, in another, DNA fingerprinting of cases of recurrent TB suggested that those previously infected with TB are at increased risk of developing TB again when re-infected [15]. This study aims to identify predictors with unfavorable outcome at the end of therapy and the long-term unfavorable outcome after treatment completion in South Korea.

## Methods

### Study population and design

Subjects in this study were recruited prospectively into an observational cohort study at the National Masan Tuberculosis Hospital and the National Medical Center in South Korea (ClinicalTrials.gov ID: NCT00341601). Both new and previously treated tuberculosis cases [defined according to World Health Organization (WHO) definitions] [17] were enrolled in this study. The inclusion criteria allowed enrollment of adults 20 years or older who had clinical signs or symptoms suggestive of TB, and had either a positive sputum smear for acid-fast bacilli or confirmed *Mycobacterium tuberculosis* in their sputum using any molecular method. In addition, new cases could not have had a treatment interruption lasting more than 60 days and must have had at least 4 months of treatment remaining in their current episode of TB. Previously treated cases must either have previously been treated for more than 30 days followed by a treatment interruption of at least 60 days or have had a history of treatment failure or chronic tuberculosis.

According to Korean tuberculosis guidelines [18], drug-resistant TB patients are treated with an individualized regimen based on results of drug-susceptibility testing. For drug-susceptible and new patients, hospital guidelines recommend a 9-month treatment duration except for cases with minimal disease that are initially sputum smear and culture negative. In this study, regular follow-up was conducted by medical chart abstraction or phone call at 3 to 6 month intervals; the final follow-up was performed up to 40 months after treatment completion. Data collected from 2005-2012 are included in this study.

### Measurements and definitions

Treatment outcomes were categorized as cure, failure, treatment interruption, unknown, death, relapse or withdrawal and were assessed at both the end of treatment (EOT) and the end of study (EOS) follow-up phone call. Treatment outcomes at EOT were defined as follows: a) cure was defined as a patient who was initially sputum smear-positive and who was sputum smear-negative in the last month of treatment and on at least one previous occasion, per the WHO Global Tuberculosis Report 2013 [2]; b) failure was defined as 6-month sputum culture positivity for drug-susceptible (DS) cases and treatment termination or need for permanent regimen change of at least two anti-TB drugs for rifampin-resistant (RR) and MDR cases; c) treatment interruption (TI) was defined as voluntary cessation of therapy for 2 or more consecutive months without restarting the same regimen within 6 months; d) death was defined as a case who died for any reason during the treatment course; e) unknown was defined as a case whose treatment outcome was not known, including loss to follow-up and lack of microbiologic information at EOT; f) withdrawal was defined as a case who withdrew from the study before treatment completion. Treatment outcomes at EOS were assessed approximately 40 months after treatment completion as follows: i) cure was defined as a case who was cured at EOT and completed the study without relapse, death, or withdrawal; ii) failure was defined as culture positivity 2 years after treatment initiation, regardless of drug-resistance; iii) death was defined as a case who died for any reason during the entire study period; iv) relapse was defined as a case who reinitiated TB treatment after being designated a cure at treatment completion.

Demographic information, including gender, age, residential area (e.g. large or small city), and socioeconomic factors, including education, occupation and housing status, were collected upon study entry. Age was categorized into three groups (20-34, 35-49, and ≥50) for the purposes of analysis. Relevant subject medical history that was collected included: alcohol consumption, smoking history, diabetes mellitus status, and previous tuberculosis treatment history, including drug-susceptibility testing (DST), elevation of total bilirubin, elevation of liver enzymes (alanine aminotransferase and aspartate aminotransferase) and chest X-ray reports. Korean tuberculosis guidelines define ‘far advanced’ disease on chest X-ray as “disseminated lesions of slight to moderate density exceeding the total volume of one lung or dense and confluent lesions exceeding one third the volume of one lung or the presence of cavities greater than 4 cm in diameter” [18]. A significant regimen change was defined as: i) any change from 1^st^-line drugs to 2^nd^-line drugs; ii) adding either a later generation fluoroquinolone or linezolid to baseline 2^nd^-line drugs; or iii) adding 2 or more classes of 2^nd^-line drugs.

### Statistical methods

Comparisons of characteristics between patients who were new and previously treated were conducted using the Mann-Whitney test for continuous variables and Fisher‘s exact test if any value was less than 5 in a cell or Pearson‘s χ^2^ test for categorical variables. The first comparison was performed to identify factors associated with unfavorable outcomes (failure or death) versus cure at EOT using logistic regression. A second comparison was conducted to identify factors associated with unfavorable outcomes (failure, death, or relapse) compared to cure at EOS by binary logistic regression. A third logistic regression was performed looking at risk factors for treatment interruptions (relative to those cured). Finally, Cox proportional hazards regression analysis was used to identify factors associated with unfavorable outcomes (death or relapse) at EOS among those cured at EOT. All multivariate models considered age, gender, and all variables that were univariately significant. With the exception of age and gender, variables that were no longer significant in the multivariate model were dropped. Statistical analyses were performed with Stata/SE 12.0 (Stata Corp., College Station, TX, USA) with *P* value < 0.05 as the criterion for statistical significance.

### Ethics

Informed consent was obtained from all participants in the study. This study was approved by the Institutional Review Boards of the National Medical Center and the National Masan Tuberculosis Hospital in South Korea, and the National Institute of Allergy and Infectious Diseases in the U.S. This study was conducted in accordance with ICH-GCP and monitored by an independent clinical research organization.

## Results

### Baseline and treatment characteristics

A total of 669 patients were enrolled in the study from 2005-2012, 563 (84.2%) of whom were male with a median age of 44 years. Except for smoking history and tuberculosis-related clinical factors, there were no significant differences in baseline characteristics between new and previously treated cases. Compared with new cases, previously treated patients presented with similar sputum smear scores but significantly more advanced and bilateral disease on baseline chest X-ray and more drug resistance (Table 1).

**Table 1:** Comparisons of baseline characteristics by new or previously treated cases (n = 669)

**Variables**		**Group (%)**		***P*-value**
		**New case**	**Previously treated case**	
***Demographic***				
Gender	Female	36 (16.1)	70 (15.7)	0.909
	Male	188 (83.9)	375 (84.3)	
Age	20-34	47 (21.0)	122 (27.4)	0.158
	35-49	95 (42.4)	183 (41.1)	
	50-	82 (36.6)	140 (31.5)	
***Health condition***				
BMI (n = 626)	<18.5	108 (50.2)	204 (49.6)	0.887
	18.5-	107 (49.8)	207 (50.4)	
Diabetes	No	180 (80.4)	340 (76.4)	0.246
	Yes	44 (19.6)	105 (23.6)	
***Individual behavior***				
Alcohol, within 6 months	Less than once a week	102 (45.5)	245 (55.1)	0.062
	Several times a week	39 (17.4)	68 (15.3)	
	At least once a day	83 (37.1)	132 (29.7)	
Smoking, within 6 months	Never smoked	56 (25.0)	125 (28.1)	<0.001
	< 1 pack/day	30 (12.39)	125 (28.1)	
	1 pack/day	78 (34.8)	121 (27.2)	
	> 1 pack/day	60 (26.8)	74 (16.6)	
***Socioeconomic***				
Residential area	Small city and town	135 (60.3)	245 (55.1)	0.199
	Large city	89 (39.7)	200 (44.9)	
Education	High school, above	120 (53.6)	251 (56.4)	0.487
	Middle school, below and refusal	104 (46.4)	194 (43.6)	
Occupation	Health care, professional, office work	16 (7.1)	55 (12.4)	0.117
	Service sector and laborer in construction or factory	128 (57.1)	242 (54.4)	
	Unemployment and others	80 (35.7)	148 (33.3)	
Housing status (n = 668)	Private	201 (89.7)	415 (93.5)	0.089
	Other	23 (10.3)	29 (6.5)	
***Tuberculosis-related clinical***				
Chest X ray				
Grade	Minimal	10 (4.5)	9 (2.0)	0.020
	Moderately advanced	111 (49.6)	187 (42.0)	
	Far advanced	103 (46.0)	249 (56.0)	
Cavity	Yes	138 (61.6)	281 (63.1)	<0.001
	No	68 (30.4)	78 (17.5)	
	Not clear	18 (8.0)	86 (19.3)	
Bilateral	Unilateral	57 (25.4)	56 (12.6)	<0.001
	Bilateral	167 (74.6)	389 (87.4)	
Nodular lesion	Yes	199 (88.8)	417 (93.7)	0.038*
	No	22 (9.8)	27 (6.1)	
	Not clear	3 (1.3)	1 (0.2)	
Treatment History				
Number of previous treatment episode (n = 444)	1	-	151 (33.9)	-
	2	-	106 (23.8)	
	3	-	63 (14.2)	
	4 or more	-	125 (28.1)	
Cumulative duration of previous treatment (days, n = 444)	Median (IQR)	-	304 (61-761)	-
History of failure (n = 445)	No	-	249 (56.0)	-
	Yes	-	196 (44.0)	
History of treatment interruption (n = 445)	No	-	207 (46.5)	-
	Yes	-	238 (53.5)	
Drug Susceptibility pattern (n = 581)	DS	146 (75.0)	92 (26.5)	<0.001*
	Mono/Poly-R	29 (14.8)	44 (11.4)	
	Rif, mono-R	4 (2.0)	16 (4.2)	
	MDR	16 (8.2)	223 (57.9)	
AFB smear score at baseline				
	-	1 (0.5)	4 (0.9)	0.689*
	Scant	51 (22.8)	85 (19.1)	
	1+	121 (54.0)	247 (55.5)	
	≥2+	51 (22.8)	109 (24.5)	

*Tested by Fisher’s exact test.

BMI: Body mass index; DS: Drug susceptible, Mono/poly-R: mono or poly-drug resistance that is not matched with the definition of Multidrug-resistant tuberculosis; MDR: multidrug-resistant tuberculosis.

By EOT, the previously treated group experienced significantly more regimen changes. Previously treated cases had lower proportions of cured and higher proportions of failed, unknown outcomes, and deaths compared to new cases (Table 2).

**Table 2:** Comparisons of treatment-course related characteristics of study population at the end of therapy by new or previously treated status (n = 669)

**Variables (n)**	**New case**	**Previously treated case**	***P*-value**
Number of significant regimen change			
0	194 (86.6)	311 (69.9)	<0.001
≥1	30 (13.4)	134 (30.1)	
Elevation of total bilirubin			
No	143 (63.8)	307 (69.0)	0.180
Yes	81 (36.2)	138 (31.0)	
Elevation of liver enzyme			
No	181 (80.8)	365 (82.0)	0.701
Yes	43 (19.2)	80 (18.0)	
Months to treatment completion, median (IQR)	9.45 (8.89-12.23)	14.97 (9.67-26.10)	<0.001†
Treatment outcome at EOT			
Cured	154 (68.8)	181 (40.7)	<0.001*
Failure	4 (1.8)	61 (13.7)	
TI	35 (15.6)	87 (19.6)	
Unknown	15 (6.7)	50 (11.2)	
Death	6 (2.7)	44 (9.9)	
Withdrawal	10 (4.5)	22 (4.9)	
Culture positivity (since enrollment)			
Converted prior to 6 months	145 (64.7)	245 (55.1)	0.001
Positivity at 6 months, over	14 (6.2)	71 (16.0)	
Unknown 6 month result	65 (29.0)	129 (29.0)	
Months to TI, median (IQR)	7.98 (5.02-10.32)	9.67 (6.60-17.83)	0.013†

*Tested by Fisher’s exact test.

† Tested by Mann-Whitney test.

AFB: Acid fast bacilli; IQR: Interquartile range; EOT: End of therapy; TI: Treatment interruption.

### Predictors of unfavorable outcomes at the end of treatment

Among the 403 patients with complete information available at EOT, 289 (71.7%) were cured, 65 failed, and 49 died. The multivariate analysis of baseline risk factors associated with unfavorable outcomes (failure or death) at EOT included diabetes (OR = 2.52; 95% CI = 1.27-5.01), patients drinking several times a week (OR = 0.38: 95% CI = 0.16-0.93, compared to drinking less than once a week), ≥1 significant regimen changes (OR = 4.01; 95%CI = 2.16-7.44), MDR-TB (OR = 2.75; 95%CI = 1.13-6.72, compared to drug-sensitive TB), and patients with 3 or ≥4 previous treatment episodes (OR = 3.28; 95% CI = 1.06-10.14 and OR = 10.30; 95% CI = 3.79-27.94, respectively, compared to no previous treatment episodes) (Table 3). The presence of cavities or bilateral lesions on chest x-ray and having one or more previous treatment failures were significantly associated with unfavorable outcomes in the univariate analysis but not in the multivariate analysis.

**Table 3:** Predictors of unfavorable outcome (compared to cure) assessed at the end of therapy (n = 403)†

**Variable**		**Univariate**		**Multivariate‡**	
		**OR**	**95% CI**	**OR**	**95% CI**
Gender	Female	1		1	
	Male	1.04	0.57-1.87	0.92	0.42-2.01
Age	20-34	1		1	
	35-49	1.09	0.64-1.85	1.00	0.48-2.09
	≥50	0.70	0.39-1.24	0.78	0.35-1.72
Body Mass Index	<18.5	1		-	
	≥18.5	0.86	0.55-1.34		
Diabetes	No	1		1	
	Yes	1.78*	1.07-2.95	2.52**	1.27-5.01
Alcohol, within 6 months	Less than once a week	1		1	
	Several times a week	0.40*	0.19-0.83	0.38*	0.16-0.93
	At least once a day	0.59**	0.36-0.98	1.10	0.56-2.19
Smoking, within 6 months	Never smoked	1		-	
	< 1 pack/day	1.11	0.63-1.94		
	1 pack/day	0.39**	0.21-0.72		
	> 1 pack/day	0.58	0.30-1.13		
Residential area	Small city and town	1		-	
	Large city	1.70*	1.10-2.64		
Education	High school, above	1		-	
	Middle school, below or refusal	0.73	0.47-1.15		
Occupation	Health care, professional and office work	1		-	
	Service sector and laborer	0.44*	0.23-0.83		
	Unemployment and others	0.56	0.29-1.08		
Chest X-ray					
Grade	Minimal or moderate	1		-	
	Far advanced	1.63*	1.05-3.54		
Cavity	No	1		-	
	Yes or not clear	1.88*	1.02-3.45		
Nodular lesion	No	1		-	
	Yes or not clear	1.48	0.59-3.76		
Bilateral	Unilateral	1		-	
	Bilateral	3.45**	1.66-7.19		
Number of previous treatment episodes	0 (new case)	1		1	
	1	2.50*	1.05-5.96	2.38	0.91-6.25
	2	5.02***	2.11-11.93	2.03	0.72-5.79
	3	10.27***	4.05-26.04	3.28*	1.06-10.14
	≥4	28.54***	13.30-61.24	10.30***	3.79-27.94
History of failure	No	1		-	
	Yes	8.00***	4.93-12.99		
History of TI	No	1		-	
	Yes	2.15**	1.37-3.38		
Number of significant regimen changes	0	1		1	
	≥1	7.26***	4.53-11.64	4.01***	2.16-7.44
Drug susceptibility pattern	DS	1		1	
	Mono/poly-R	2.40	0.93-6.18	0.93	0.31-2.78
	Rif, mono-R	2.74	0.53-14.00	0.58	0.07-4.46
	MDR	14.18***	7.47-26.90	2.75*	1.13-6.72

*p <0.05, **p <0.01, ***p < 0.001.

† Favorable outcome is cured (n = 289) and unfavorable outcome includes failure (n = 65) and death (n = 49) assessed at the end of therapy.

‡ Multivariate model includes age, gender and all significant factors from the univariate model.

TI: treatment interruption; DS: Drug susceptible, Mono/poly-R: mono or poly-drug resistance that is not matched with the definition of multidrug-resistant tuberculosis or rifampin mono resistance; MDR: multidrug-resistant tuberculosis.

### Long-term predictors of unfavorable outcomes at the end of study

Among the 392 patients followed to EOS (40 months after EOT), 267 (67.6%) were considered cures, 22 failed, 17 relapsed, and 86 died. The multivariate analysis of baseline risk factors associated with unfavorable outcomes (failure, relapse, or death) at EOS included age 35-49 or age ≥50 (OR = 2.14; 95% CI = 1.11-4.14 and OR = 2.97; 95% CI = 1.51-5.86, respectively, compared to those age 20-34), diabetes (OR = 2.57; 95% CI = 1.46-4.52), and having MDR-TB (OR = 4.51, 95% CI = 2.64-7.68, compared to drug sensitive TB) (Table 4). Having a body-mass index (BMI) ≥18.5 was associated with cure (OR = 0.33; 95% CI = 0.20-0.54, compared to BMI <18.5). Baseline chest x-ray status and previous treatment history were significantly associated with unfavorable outcomes in the univariate analysis but not in the multivariate analysis.

**Table 4:** Predictors of unfavorable outcome (compared to cure) assessed at the end of study (n = 392) †

**Variable**		**Univariate**		**Multivariate‡**	
		**OR**	**95% CI**	**OR**	**95% CI**
Gender	Female	1		1	
	Male	1.29	0.70-2.35	1.14	0.59-2.20
Age	20-34	1		1	
	35-49	1.96*	1.07-3.57	2.14*	1.11-4.14
	≥50	2.75**	1.50-5.05	2.97**	1.51-5.86
Body Mass Index	<18.5	1		1	
	≥18.5	0.47**	0.31-0.73	0.33***	0.20-0.54
Diabetes	No	1		1	
	Yes	2.47***	1.51-4.05	2.57**	1.46-4.52
Alcohol, within 6 months	Less than once a week	1		-	
	Several times a week	0.52	0.26-1.05		
	At least once a day	0.92	0.58-1.48		
Smoking, within 6 months	Never smoked	1		-	
	< 1 pack/day	1.71	0.99-2.97		
	1 pack/day	1.14	0.66-1.97		
	> 1 pack/day	1.03	0.57-1.89		
Residence	Small city and town	1		-	
	Large city	1.19	0.78-1.83		
Education	High school, above	1		-	
	Middle school, below or refusal	1.40	0.92-2.15		
Occupation	Health care, professional and office work	1		-	
	Service sector and laborer	1.35	0.66-2.76		
	Unemployment and others	1.54	0.73-3.24		
Chest X-ray					
Grade	Minimal or moderate	1		-	
	Far advanced	2.09**	1.35-3.24		
Cavity	No	1		-	
	Yes or not clear	1.25	0.72-2.15		
Nodular lesion	No	1		-	
	Yes or not clear	1.44	0.60-3.48		
Bilateral	Unilateral	1		-	
	Bilateral	2.67**	1.38-5.18		
Number of previous treatment episodes	0 (new case)	1		-	
	1	1.7	0.90-3.22		
	2	1.04	0.48-2.27		
	≥3	4.66***	2.69-8.08		
History of failure	No	1		-	
	Yes	3.32***	2.11-5.22		
History of TI	No	1		-	
	Yes	2.26***	1.45-3.51		
Number of significant regimen changes	0	1		-	
	≥1	1.21	0.75-1.93		
Drug Susceptibility pattern (n = 401)	DS	1		1	
	Mono/poly-R	1.42	0.68-2.97	1.45	0.67-3.13
	Rif, mono-R	1.69	0.42-6.87	2.87	0.64-12.95
	MDR	3.39***	2.09-5.50	4.51***	2.64-7.68

*p <0.05, **p <0.01, ***p < 0.001.

† Favorable outcome is cured (n = 267) and unfavorable outcome includes failure (n = 22), death (n = 86) and relapse (n = 17) assessed at the end of study.

‡ Multivariate model includes age, gender and all significant factors from the univariate model.

TI: treatment interruption; DS: Drug susceptible, Mono/poly-R: mono or poly-drug resistance that is not matched with the definition of multidrug-resistant tuberculosis or rifampin mono resistance; MDR: multidrug-resistant tuberculosis.

### Predictors of treatment interruption (TI) at the end of therapy

Among the total 457 patients with full information at EOT, 335 were cured and 122 experienced a TI. In the multivariate analysis compared with those who were cured, those with a TI were significantly more likely to work in the service sector or as a laborer (OR = 2.30; 95% CI = 1.36-3.90, compared with those unemployed), present with bilateral lesions on chest X-ray (OR = 2.46; 95% CI = 1.22-4.95), or have a previous treatment failure (OR = 1.76; 95% CI = 1.04-2.98) or previous TI (OR = 2.35; 95% CI = 1.49-3.70) (Table 5).

**Table 5:** Predictors of treatment interruption (compared to cures) (n = 457) †

**Variable**		**Univariate**		**Multivariate‡**	
		**OR**	**95% CI**	**OR**	**95% CI**
Gender	Female	1		1	
	Male	1.48	0.79-2.78	1.53	0.78-3.00
Age	20-34	1		1	
	35-49	1.16	0.69-1.95	0.92	0.53-1.59
	≥50	0.67	0.38-1.18	0.63	0.34-1.16
Body Mass Index (n = 446)	<18.5	1		-	
	≥18.5	0.78	0.51-1.91		
Diabetes	No	1		-	
	Yes	1.56	0.95-2.56		
Alcohol, within 6 months	Less than once a week	1		-	
	Several times a week	1.71	0.96-3.05		
	At least once a day	2.03**	1.27-3.24		
Smoking, within 6 months	Never smoked	1		-	
	< 1 pack/day	2.68**	1.31-5.49		
	1 pack/day	3.36***	1.75-6.46		
	> 1 pack/day	3.12**	1.54-6.32		
Residence	Small city and town	1		-	
	Large city	1.20	0.79-1.83		
Education	High school, above	1		-	
	Middle school, below or refusal	1.20	0.80-1.82		
Occupation	Unemployment and others	1		1	
	Health care, professional and office work	1.14	0.47-2.75	0.89	0.35-2.25
	Service sector and laborer	2.39**	1.45-3.95	2.30**	1.36-3.90
Chest X-ray					
Grade	Minimal or moderate	1		-	
	Far advanced	1.34	0.89-2.04		
Cavity	No	1		-	
	Yes or not clear	1.04	0.63-1.69		
Nodular lesion	No	1		-	
	Yes or not clear	2.07	0.70-6.14		
Bilateral	Unilateral	1		1	
	Bilateral	2.86**	1.46-5.60	2.46*	1.22-4.95
Number of previous treatment episode	0 (new case)	1		-	
	1	1.54	0.87-2.71		
	2	2.77**	1.53-5.02		
	≥3	2.40**	1.35-4.27		
History of failure	No	1		1	
	Yes	1.81*	1.12-2.92	1.76*	1.04-2.98
History of TI	No	1		1	
	Yes	2.72***	1.77-4.18	2.35***	1.49-3.70
Elevation of total bilirubin	No	1			
	Yes	0.63*	0.40-1.00		
Elevation of liver enzyme	No	1			
	Yes	0.95	0.55-1.65		
Number of significant regimen changes	0	1		-	
	≥1	1.62*	1.04-2.55		
Drug susceptibility pattern (n = 403)	DS	1		-	
	Mono/poly-R	1.25	0.66-2.39		
	Rif, mono-R	2.01	0.68-5.92		
	MDR	1.45	0.88-2.39		

*p <0.05, **p <0.01, ***p < 0.001.

†Treatment interruption (n = 122) was compared with cured (n = 335).

‡Multivariate model includes age, gender and all significant factors from the univariate model.

TI: Treatment interruption, DS: Drug susceptible, Mono/poly-R: mono or poly-drug resistance that is not matched with the definition of multidrug-resistant tuberculosis or rifampin mono resistance; MDR: multidrug-resistant tuberculosis.

### Long-term prognosis of cured cases at the end of therapy

Among the total 289 cured cases with full information at EOT, 278 (96.2%) cases had full follow-up information to EOS. After treatment completion, there were 20 deaths and 17 relapse cases with full baseline and follow-up information. Age ≥50, BMI and education were associated with death or relapse in univariate analysis. In the multivariate analysis, the factors that remained significant were age ≥50 (OR = 3.11; 95% CI = 1.17-8.26, compared to those age 20-34) and BMI ≥18.5 being associated with cure (HR = 0.49; 95% CI = 0.25-0.95). Having MDR-TB at baseline was not a significant prognostic factor in univariate analysis (HR = 0.87; 95% CI = 0.40-1.89; not shown in table) (Table 6).

**Table 6:** Prognostic factors of unfavorable outcome at end of study among cured cases (n = 278) †

	**Adjusted HR‡**	**95% CI**
*Age*		
20-34	1	
35-49	1.38	0.48-3.98
≥50	3.11*	1.17-8.26
*Gender*		
Female	1	
Male	1.14	0.44-2.95
*Body Mass Index*		
<18.5	1	
≥18.5	0.49*	0.25-0.95

*p <0.05.

†Among cases who had full information at the end of study, cured cases (n = 241) were compared with death (n = 20) and relapse (n = 17) cases.

‡Multivariate model includes age, gender and all significant factors from the univariate model (omitted in this paper).

TI: Treatment interruption, DS: Drug susceptible, Mono/poly/Rif-R: mono or poly-drug resistance that is not matched with the definition of multidrug-resistant tuberculosis; MDR: multidrug-resistant tuberculosis.

## Discussion

Multiple previous cohort studies have reported extensively on the risk factors of tuberculosis outcomes, using various measures including relapse [15,16], mortality [7,10,14,19], MDR or XDR-TB status [11,13,20], and treatment failure, TI, or death [12,21,22]. In addition, differing study end points were used, including the end of therapy [12,21,22], long term follow up after treatment completion [14-16], and cross-sectional comparison between M/XDR and potential risk factors [11,13,20]. Our study identified factors associated with poor outcomes among all patients at EOT and after long-term follow-up at EOS. In addition, in an effort to separate EOS risk factors from EOT risk factors, an analysis was done to identify factors for relapse or death at EOS just among those considered cured at EOT. At EOT, having diabetes, ≥3 previous treatment episodes, ≥1 significant regimen changes, and MDR-TB were all significantly associated with treatment failure or death (Table 3). At EOS, having diabetes and MDR-TB at baseline continued to be significantly associated with treatment failure, death, or relapse. In addition, baseline age ≥35 and BMI <18.5 were also poor prognostic factors, but the number of previous treatment episodes was no longer significant (Table 4). These risk factors, including age, BMI, and drug resistance pattern, are consistent with those of a previous study with long-term follow-up [14]. These results may suggest that older age and low BMI contribute to relapse or death risk after treatment completion. In isolating risk factors significantly associated with poor outcomes at EOS among cures at EOT, only age ≥50 and baseline BMI <18.5 were identified (Table 6), suggesting that once a patient is cured, many traditional baseline predictive variables, such as chest x-ray status, previous TB treatment history, and MDR-TB status may no longer be as relevant for prognosis. A different study analyzed factors for TB recurrence at EOS after a median 4-year follow-up after EOT and identified inner-city residence, HIV infection, and history of TB treatment as risk factors [16]. This study, however, included all treatment-completed patients at EOT regardless of culture conversion status and thus included some patients not fully cured. Future relapse studies among cured patients should be conducted to understand if their risk factors revert back to those similar to the general population.

Some previous studies included TI as an unfavorable outcome (24-26). Because our study population had a relatively high proportion of TI (18.2%) and reasons for TI may be very different from reasons for failure, death, or relapse, we analyzed this outcome separately, comparing TI cases only to cured cases. Other studies have also identified risk factors associated specifically with treatment default, including long distance to health facilities, substance use including alcohol consumption, a fear of social stigmatism, unemployment and economic constraint [10,19,23-25]. In our analysis, alcohol consumption and smoking history were associated with TI in the univariate analysis but the significance was lost in the multivariate analysis. Not surprisingly, our study also identified an increased risk for TI among those who previously had a TI or treatment failure. For unclear reasons, having bilateral disease on baseline chest x-ray was associated with TI. Finally, being a blue-collar worker was also risk factor for TI, compared to those who were unemployed. This suggests that perhaps low wage workers had more difficulty getting time off for medical care and may require healthcare policy changes to resolve this issue. Further studies may need to be done to understand better the reasons for TI among service sector employees and laborers. Understanding the causes of TI and how to reduce treatment default is critically important because of the association of TI with the development of drug resistance [26].

Our study has several limitations. First, the study was conducted at two tertiary referral hospitals, which usually manage patients with more severe and extensive disease. This is reflected in our cohort demographics, with most patients having more severe advanced disease on x-ray, including cavities and bilateral disease, as well as the proportion of previously treated cases (compared to new cases) and MDR-TB. Our results, therefore, may not be representative of patients with less severe disease. Second, we only had DST results for 581/669 (86.8%). Analyses that include DST results exclude patients without DST results, which may cause a selection bias and the point estimates may be over or under-estimated due to the excluded cases. Third, we were not able to measure certain factors possibly related to treatment outcome, including hemoglobin A1c and bacterial load (time to positivity in liquid culture system). Fourth, diabetes status collected by self-report and types I vs. II could not be differentiated. Finally, the study was not designed to identify a causal pathway between the independent and outcome variables and was limited to identifying factors associated with unfavorable outcomes and TI.

## Conclusions

This study of risk factors associated with poor treatment outcomes at EOT and EOS highlights both patient specific factors that are difficult to change, such as diabetes status and age, as well as disease specific factors, such as multidrug resistance, that may be affected by programmatic factors and could be altered to improve prognosis. Risk factors related to prolonged disease at diagnosis, including history of multiple previous treatment episodes, emphasize that the initial treatment episode is also likely the best chance of cure. Program management changes should be considered to emphasize public health measures such as directly observed therapy to help ensure that the first treatment episode is also the last one, in conjunction with improving adherence and decreasing the risk of MDR-TB. Policy changes to enable all TB patients, regardless of work status, to have time off to get appropriate treatment would also be important. Finally, steps to improve nutrition and therefore BMI among those successfully treated may help prevent longer-term relapses or recurrences. The treatment of tuberculosis requires a multifaceted approach for the best chance of success.

## Competing interests

The authors declare that they have no competing interests.

## Authors’ contributions

HC, ML, TS, YC, LCG, LEV, MWC, SNC, and CEB designed the study. HC, ML, YK. SY, JSJ, and SKP conducted the study and collected the data. JL and TS conducted the laboratory analyses. HC, ML, RYC, and LED analyzed and interpreted the data. HC, ML, and RYC drafted the manuscript. All authors read and approved the final manuscript.

## Pre-publication history

The pre-publication history for this paper can be accessed here:

http://www.biomedcentral.com/1471-2334/14/360/prepub
